# The Impact of Autosomal Dominant Polycystic Kidney Disease in Children: A Nephrological, Nutritional, and Psychological Point of View

**DOI:** 10.3390/biomedicines12081823

**Published:** 2024-08-12

**Authors:** Matteo Guarnaroli, Flavia Padoan, Cristiano Fava, Maria Giulia Benetti, Milena Brugnara, Angelo Pietrobelli, Giorgio Piacentini, Luca Pecoraro

**Affiliations:** 1 Pediatric Unit, Department of Surgical Sciences, Dentistry, Gynecology and Pediatrics, University of Verona, 37126 Verona, Italy; 2 General Medicine and Hypertension Unit, Department of Medicine, University of Verona, 37126 Verona, Italy; cristiano.fava@univr.it

**Keywords:** ADPKD, kidney cystic disease, ADPKD-related cardiovascular complications, ADPKD-related nutrition issue, ADPKD-related psychological issue, multidisciplinary follow-up

## Abstract

Autosomal dominant polycystic kidney disease (ADPKD) is a hereditary disorder characterized by the formation of numerous fluid-filled cysts in the kidneys, leading to progressive renal failure and various extrarenal complications, including hypertension. This review explores the genetic basis of ADPKD, including emerging evidence of epigenetic mechanisms in modulating gene expression and disease progression in ADPKD. Furthermore, it proposes to examine the pathological characteristics of this condition at the nephrological, cardiovascular, nutritional, and psychological levels, emphasizing that the follow-up of patients with ADPKD should be multidisciplinary from a young pediatric age.

## 1. Introduction

Autosomal dominant polycystic kidney disease (ADPKD) is the most common genetic renal disorder [1]. It affects 12.5 million people worldwide, with no differences in prevalence across ethnicities or genders [2]. However, male patients appear to experience a more rapid progression of the disease [3]. Typically, ADPKD remains asymptomatic until adulthood, although in a small percentage of cases, it can present with rapidly progressive renal failure from childhood. The disease is characterized by progressive cystic degeneration of the kidneys and other parenchymal organs, along with extrarenal manifestations, particularly cardiovascular abnormalities. ADPKD involves the bilateral and progressive development of cysts, primarily in the kidneys but less frequently in other locations such as the liver, pancreas, seminal vesicles, and subarachnoid space [4]. Throughout the disease, the number of cysts progressively increases with age. In the majority of affected individuals under the age of 30, only a few unilateral or bilateral cysts are typically observed. However, hundreds to thousands of cysts can be found in both kidneys by the fifth decade of life, increasing renal size [5]. The proliferation of cysts and the increase in renal volume lead to a loss of organ function, eventually causing end-stage renal disease (ESRD). This condition requires renal replacement therapy or organ transplantation in 50% of cases by the age of 60 [6].

ADPKD falls within a heterogeneous group of disorders known as ciliopathies. The development of these cysts is genetically determined and attributed to alterations in the structure and functional activity of the primary cilium, a non-motile organelle present on the surface of many human cells, including renal cells. In the kidneys, the primary cilium regulates the proliferation of tubular epithelial cells, acts as a mechanosensor for detecting urine flow within the tubular lumen, modulates the reabsorption of urinary solutes, and presents ligands in the urine to membrane receptors [7]. ADPKD is inherited in an autosomal dominant manner. The genes involved are *PKD1*, which encodes Polycystin 1 (PC1), and *PKD2*, which encodes Polycystin 2 (PC2). In 72–75% of resolved ADPKD cases, a mutation is found in the *PKD1* gene, while 15–18% have a mutation in the *PKD2* gene. A single mutated allele is sufficient to manifest the disease, and in 95% of cases, this allele is inherited from one of the parents [8]. PC1 and PC2 are located on the membrane and cilia of renal tubular epithelial cells throughout the individual’s life, starting from zygote development. PC1 is an integral membrane glycoprotein functioning as a G-protein coupled receptor (GPCR), while PC2 forms a non-selective cation channel permeable to calcium ions that interact with PC1 [9]. Approximately 7–10% of ADPKD cases are genetically unresolved (GUR), with the main gene involved being GANAB, which encodes the catalytic subunit of Glucosidase II (GIIα), a protein necessary for the maturation and localization of PC1 and PC2. Loss of GIIα function is associated with a phenotype characterized by a few large cysts that increase kidney size without progressing to ESRD [10,11]. The PC1/PC2 complex develops from the interaction between PC1 and PC2 within the endoplasmic reticulum. Subsequently, it migrates to the primary cilium or the plasma membrane at the cell–matrix or cell–cell interface [12]. This complex mediates cell–cell and cell–extracellular matrix interactions. The PC1/PC2 complex plays a role in the cell cycle, modulating the JAK–STAT pathway to arrest the cell cycle in G0 and prevent cell proliferation [13]. Additionally, PC2 interacts with PC1 to form a non-selective cation channel that facilitates calcium influx into the endoplasmic reticulum. Specifically, PC1, located on the primary cilium, is a mechanoreceptor [14]. Following deformations caused by urinary flow, the cilium functions as a sensory transducer, increasing the intracellular Ca++ flow through the opening of the Ca++ channel by the PC1/PC2 complex. A pathogenic mutation in the *PKD1* or *PKD2* genes leads to the inactivation or reduction of the PC1–PC2 complex activity, resulting in decreased intracellular calcium and calcineurin concentrations, subsequently affecting target gene transcription. Furthermore, low calcium concentrations activate adenylate cyclase, accumulating cyclic AMP (cAMP), which regulates the tubular secretion of Cl− [15]. The loss of function of the PC1/PC2 complex results in a poorly differentiated, hyperproliferative cellular phenotype that is no longer absorptive but secretory, leading to the development of renal cysts [16,17]. In ADPKD, cysts can originate from any nephron segment and lose communication with the tubule, distinguishing them from the typical cysts of ARPKD, which derive from the collecting duct and maintain communication with it [18]. Despite all nephron cells harboring the same germline mutation in ADPKD, only 5–10% of nephrons develop cysts, giving the disease a focal characteristic. The genetic mechanism underlying the focal process of ADPKD remains a subject of discussion. The “two-hit” model is the most widely accepted among the proposed hypotheses. The initial event in cystogenesis is the loss of somatic heterozygosity in the *PKD1* or *PKD2* genes. The transmission of the mutated gene from one parent requires a second mutation in the individual’s somatic line, as hypothesized in the “two-hit” model. Somatic mutations are rare and occur in a relatively limited number of cells, contributing to the focal nature of cyst distribution in ADPKD [19]. Each renal cyst originates from a “second hit,” meaning that many mutations are necessary for the disease manifestation in polycystic kidney disease. It is estimated that renal tissue has a somatic mutation rate approximately ten times higher than other tissues [20]. Therefore, in the “two-hit” model, inheriting a single mutated gene copy is insufficient; a second somatic mutation is required to trigger cyst formation. However, it has been demonstrated that complete inactivation of *PKD1* or *PKD2* copies is unnecessary to initiate cystogenesis. Cyst formation occurs with a low level of polycystin (around 20%), a concept known as the “threshold model” [21]. The threshold model encompasses the “two-hit” model as one of the main causes but not the only explanation. Variants in other PKD-related genes, unidentified modifier genes, and environmental factors such as acute kidney injury can modulate cyst formation and disease progression, increasing the likelihood of cystogenesis as PC1 and PC2 levels drop below threshold levels [21,22]. It has been hypothesized that a third event (such as a transient renal tubule obstruction or an ischemic event) may cause epithelial damage that cannot be repaired due to polycystin mutations. The renal tissue, unable to repair, undergoes hyperproliferation, leading to cyst formation. This model is referred to as the “third-hit model”. In ADPKD patients, subclinical acute kidney injuries may contribute to rapid disease progression and a more severe phenotype [23]. Another group has described the “snowball effect”, where local phenomena arising in a single cyst promote the dilation of surrounding tubules, which can evolve into a larger cyst or a cluster of cysts, highlighting how local events can contribute to the individual phenotype [24]. The penetrance of ADPKD is complete; however, its phenotypic expression, both within and among families, is highly variable [8]. Intra- and interfamily clinical variability can be explained by various factors. The first is the type of involved gene and the locus variability. Individuals with *PKD1* mutations exhibit more severe renal disease characterized by a higher number of renal cysts, early-onset hypertension, and ESRD almost two decades earlier, with lower patient survival rates. Another aspect to consider, highlighted by the GENKYST study, is allelic variability related to the type and location of mutations. This study demonstrated that the mutation type in the *PKD1* gene was strongly correlated with renal survival. In contrast, the mutation location, as shown in previous studies, was not: the median age of ESRD onset in patients with truncating *PKD1* mutations (frameshift, nonsense, or large rearrangements) was significantly different (55.6 years) from that in patients with non-truncating mutations (missense mutations) (66.7 years) [25]. Other studies have also suggested that some *PKD1* missense mutations act as hypomorphic alleles and are associated with milder forms of ADPKD [26,27]. There is also a genotype–phenotype relationship depending on gender. It has been observed that males with *PKD1* mutations have lower renal survival than females with the same mutation type (the median age of ESRD onset is 56.1 years for males versus 59.5 years for females). Finally, part of the variability is due to other genetic and environmental factors, including hypomorphic mutations, trans-heterozygous mutations, mosaicism, and modifier genes. An allele is considered hypomorphic when it produces a reduced effect compared to the wild-type allele. Rossetti et al. were the first to describe two consanguineous families segregating hypomorphic mutations in *PKD1*. Individuals with a single mutation had a mild form of the disease. In contrast, those inheriting both mutated alleles in homozygosity had a typical or severe form of the disease [8,28]. Hypomorphic alleles are found in patients with a mild form of the disease and healthy individuals. However, Rossetti et al. demonstrated that they could cause severe phenotypes and early disease onset if present in conjunction with a pathogenic variant [29]. Additionally, it has been shown that complete inactivation of both alleles is optional, because cystogenesis can be triggered even by lower levels (10–20%) of wild-type PC1 protein. Hypomorphic alleles can contribute to this phenomenon, resulting in milder clinical phenotypes in ADPKD patients with mutations in the *PKD1* gene [26]. The second factor influencing clinical variability in ADPKD is trans-heterozygosity, which is double heterozygosity in two mutations on two different genes. In 2000, Koptides et al. observed that not all cysts analyzed by biopsy in patients were caused by the sole loss of function of the wild-type allele of *PKD1* in the germline but that this mutation was associated with a somatic mutation in *PKD2* [30]. A comprehensive mutational screening, including *PKD1*, *PKD2*, *HNF1β*, and other genes, may partly explain the clinical variability of the disease within the same family. Mosaicism is another factor that may influence clinical variability, where an individual harbors at least two cellular populations with different genetic makeup. Mosaicism could be the cause of ADPKD in patients with a silent family history and may explain the presence of the disease when mutations in *PKD1* and *PKD2* are not detected [31]. Lastly, another complexity factor characterizing the disease is the presence of phenocopies and mutations in modifier genes. Phenocopies are cystic pathologies clinically mimicking ADPKD but determined by different genotypes. Mutations in genes other than *PKD1* and *PKD2*, such as HNF1β, PKHD1, or TSC2, can mimic the clinical picture of ADPKD. For example, mutations in the *HNF1β* gene may lead to various phenotypes characterized by renal cysts and diabetes (RCAD syndrome) [32]. On the other hand, mutations in the *PKHD1* gene can lead to the development of the recessive form of autosomal recessive polycystic kidney disease (ARPKD), typically starting in infancy [33]. Lastly, mutations in TSC2 can result in a syndrome characterized by very early-onset ADPKD and clinical manifestations typical of tuberous sclerosis [34]. The *TSC2* gene locus is located on chromosome 16, close to the *PKD1* locus. Therefore, a large chromosomal deletion involving both genes causes this contiguous gene syndrome. In rare cases, the cystic phenotype may be influenced by other complex ciliopathies such as Bardet–Biedl syndrome, Joubert syndrome, and Meckel syndrome [35]. There are also forms of cystic renal dysplasia that do not involve ciliary genes. In 2007, the PKDB (Autosomal Dominant Polycystic Kidney Disease Mutation Database) was developed [36]. This database contains all pathogenic germline and somatic variants, polymorphisms, and indeterminate variants of unknown significance (GUR) in the *PKD1* and *PKD2* genes. The rationale behind this database stems from the fact that no hot spots have been identified, and the mutations identified so far in the studied families are almost private mutations, rarely shared by different families.

### 1.1. Renal Manifestations

ADPKD is typically asymptomatic in pediatric age. The diagnosis of ADPKD generally occurs in adulthood. In children, the diagnosis is often linked to a suggestive family history or the incidental discovery of cysts during an ultrasound examination. Neonatal ADPKD is exceptionally rare and manifests at birth or within the first few weeks of life. Unlike the more common adult form, neonatal ADPKD is frequently associated with significant renal impairment and other complications from the outset. A positive ultrasound for a renal cyst in a patient with a family history of ADPKD can be considered diagnostic, given the rarity of simple cysts in pediatric patients. However, ultrasound cannot exclude ADPKD in the absence of cysts in patients under 40 [37]. In patients whose diagnosis is made based on a family history of ADPKD and renal cysts, genetic testing is not currently indicated as it does not add any useful information to treatment. Further genetic investigation is recommended in cases of fetuses with multiple unilateral cysts associated with extrarenal manifestations (suspected malformation pathology) and/or bilateral cysts or hyperechogenic and enlarged kidneys (especially if oligohydramnios and extrarenal manifestations are present) [38]. Regarding asymptomatic pediatric patients with a family history of ADPKD, the diagnosis poses an ethical dilemma. Early diagnosis of an incurable genetic condition has significant implications for the quality of life of an otherwise healthy individual for many years. The diagnosis allows the implementation of strategies to slow disease progression, such as a low-sodium diet, increased fluid intake, antihypertensive therapy, and antiproteinuric therapy. Less frequently, children may present with hematuria or proteinuria. Other manifestations, whose frequency, however, does not seem significant compared to the general population, include flank pain, enuresis, urinary frequency (potentially related to glomerular hyperfiltration), and urinary tract infections [39]. ADPKD is primarily characterized by the slow but constant cystic dilation of renal tubules, leading to compression of the remaining renal parenchyma and progressive renal enlargement. The hyperfiltration of unaffected nephrons (creatinine clearance ≥ 140 mL/min/1.73 m^2^) helps maintain renal function for many years. However, there is a gradual decline in renal function, eventually progressing to ESRD at a highly variable age due to the conditions above [40]. Clinical studies correlate glomerular hyperfiltration with efferent arteriole vasoconstriction caused by angiotensin II [41]. This hyperfiltration leads to a progressive increase in kidney size [42], making total kidney volume (TKV) a potential early indicator of organ damage [43]. In pediatric ADPKD patients, given the smaller size of the kidneys, TKV measured by ultrasound shows a good correlation with TKV measured by magnetic resonance imaging (MRI) [44]. In families with mutations causing manifestations in childhood, the enlargement of renal mass can be observed within the same family, presenting with varying degrees of severity [45]. About 2–5% of patients exhibit early onset with rapidly progressive renal insufficiency, leading to ESRD in childhood. The CRISP study has shown that in adult patients, the decline in GFR follows an increase in TKV by approximately 3–5 years. For every increase of 100 cm^3^/m in TKV, the likelihood of progressing to CKD stage 3 increases by 1.48 times over eight years. There are currently no comparable data for pediatric patients [46]. Acute or chronic pain in the dorsal or lumbar region can be a typical manifestation of the onset of cystic kidney disease, resulting from either the stretching of the renal capsule due to cyst enlargement or a cyst hemorrhage of spontaneous or traumatic origin. In 35–50% of individuals with ADPKD, the initial manifestation can be hematuria, mostly macroscopic. In pediatric patients with ADPKD, this hematuria may follow intense physical activity or trauma. Hematuria can also result from renal lithiasis, infection, spontaneous cyst hemorrhage, or, after the age of 50, from urothelial or renal carcinoma. Pain remains the primary symptom in polycystic kidney disease, while hematuria is secondary, as the vast majority of cysts are not in continuity with the urinary tract [42]. Regarding proteinuria (>300 mg/day), it occurs in 25% of adult cases and can originate at the tubular or glomerular level. Proteinuria influences prognosis and progression toward ESRD [39]. Proteinuria is associated with a more aggressive pathology characterized by kidney enlargement and reduced creatinine clearance [45]. Another manifestation of polycystic kidney disease is a deficit in urinary concentration, which can also be an initial symptom and may lead to polyuria and polydipsia in some cases [47]. This concentration deficit, diagnosable with a nocturnal dehydration test, results in renal water loss not compensated by fluid intake, prompting a hyperosmotic stimulus to secrete vasopressin and activate the sympathetic nervous system. Additionally, children with ADPKD have a higher incidence of urolithiasis compared to their peers. Urolithiasis can be favored by urinary stasis caused by the anatomical disruption of the kidney, decreased urinary ammonium excretion, reduced urinary pH, and hypocitraturia. The composition of these stones is primarily of uric acid, with calcium oxalate being less common [41]. Assessing the progression of ADPKD in children presents unique challenges due to the limited availability of biomarkers that are effective in adults. In adults, several biomarkers, such as plasma copeptin, urinary MCP-1 (monocyte chemoattractant protein-1), and urinary EGF (epidermal growth factor), have been correlated with disease severity and progression. Plasma copeptin is a surrogate marker for endogenous vasopressin levels, providing insights into renal function and disease activity [48]. Urinary MCP-1, a chemotactic factor for monocytes, reflects inflammation and macrophage activation within the kidneys [49,50,51], while urinary EGF, a marker for tubular cell mass, indicates renal function and progression in chronic kidney disease (CKD) [52,53]. However, these biomarkers have yet to be extensively studied in the early stages of ADPKD, particularly in pediatric populations. This represents a significant gap in research, as the progression of ADPKD can vary greatly between individuals, and early markers are crucial for timely intervention and management. Children with ADPKD often present with different disease manifestations compared to adults, potentially requiring age-specific biomarkers to monitor disease progression accurately [54].

### 1.2. Cardiovascular Manifestations

Hypertension is the most frequent manifestation of ADPKD in pediatric patients [55]. As previously mentioned, proteinuria, particularly microalbuminuria, is described in 30–48% of pediatric patients, although there is no reported association between this condition and the onset of hypertension in the literature [56]. However, evidence suggests that compensatory mechanisms, such as hypersecretion of vasopressin in response to urinary concentration deficit, may contribute to the development of hypertension in the long term [57]. In the pediatric population, arterial hypertension is observed in 20% of cases and tends to be higher in very early onset (VEO) patients. These patients are diagnosed with ADPKD before 18 months of age due to factors such as impaired renal function, oligohydramnios, larger kidney size, and hypertension [58]. This prevalence was extrapolated from a significant meta-analysis published in 2016, which included data from 928 children with a clinical diagnosis of ADPKD [59]. However, measurement methods were not uniform across studies, with most using office blood pressure (BP) measurements. The use of 24 h ambulatory blood pressure monitoring (ABPM) reveals an increased diagnosis of hypertension in ADPKD patients compared to office measurements, with a high proportion of children exhibiting isolated nocturnal hypertension [55]. These data emerged from a multicenter retrospective study published in 2018, ADPKiDs, which collected ABPM data from 310 ADPKD patients under 18 in Europe. The prevalence of hypertension in children on antihypertensive medication was 31%, 42%, and 35% during the day, night, and entire 24 h period, respectively. Additionally, 52% of patients did not exhibit physiological nocturnal dipping, and 18% had isolated nocturnal hypertension [55]. However, this study may present a potential bias due to its retrospective nature, selecting only patients with previously detected office hypertension who were then indicated for ABPM. One-fifth of the patients were on antihypertensive therapy at the time of measurement. Other studies in children with ADPKD correlate hypertension with TKV. Although it is not yet definitively established which of the two is the initiating factor, there remains a strong correlation between these two factors, with larger cyst volumes and TKV observed in patients with hypertension [60]. The pathogenesis of arterial hypertension in ADPKD patients is multifactorial [55], and unlike other nephrological conditions, hypertension appears before the loss of renal function [61]. Immunohistochemical studies on nephrectomized kidneys of ADPKD subjects have shown hyperplasia of renin-secreting cells in the juxtaglomerular apparatus [58], suggesting chronic stimulation by the renin–angiotensin–aldosterone system (RAAS). This hypothesis is supported by high renin levels, angiotensin-converting enzyme (ACE), angiotensinogen, and angiotensin II in the fluid within renal cysts [62]. Evidence indicates that the enlargement of renal cysts compresses small intrarenal vessels, causing ischemic damage and subsequently activating the RAAS [63]. Comparative studies between ADPKD patients with hypertension and normal renal function and those with essential hypertension have shown higher levels of plasma aldosterone and renin in ADPKD subjects in orthostatic and clinostatic positions, and after ACE-inhibitor (Captopril) stimulation [58]. This suggests that intrarenal RAAS activation plays a predominant role in causing hypertension compared to systemic circulatory mechanisms (e.g., nitric oxide (NO) synthesis). Moreover, higher levels of circulating vasopressin in hypertensive ADPKD patients than in hypertensive non-ADPKD patients may also contribute to hypertension [64]. Additionally, increased sympathetic nervous system activity, which stimulates RAAS and vice versa, has been demonstrated [62]. Angiotensin II contributes to the development and growth of renal cysts through vascular hypertrophy and consequent ischemia of the surrounding tissue. Activated RAAS promotes aldosterone secretion, which, in turn, enlarges cysts by retaining Na+ and drawing water into the cysts. Aldosterone also activates the epidermal growth factor receptor (EGFR), which increases the growth of undifferentiated renal tubular cells [39] and, along with angiotensin II, stimulates fibroblast growth through TGF-beta enhancement, leading to fibrosis of the tubule wall and renal tissue [65]. Furthermore, hypertension in ADPKD patients is associated with endothelial dysfunction and NO deficiency, leading to vascular remodeling. Endothelial alteration is favored by increased levels of endothelin-1 (a vasoconstrictor) in the cyst epithelium [65], cyst fluid, and plasma [66], along with reduced NO synthase activity [64], resulting in decreased NO production (a vasodilator). In ADPKD, the *PKD1* or *PKD2* gene defect leads to a calcium-dependent reduction in NO formation. Additionally, in ADPKD patients, the expression of mutated polycystins [62] in the endothelium and vascular smooth muscle is implicated in causing hypertension and renal vascular remodeling independently of RAAS [55]. The reduced activity of mutated polycystins decreases intracellular, intraplasmic, and intra-articular calcium and increases cAMP and adenylate cyclase, resulting in endothelial dysfunction. This dysfunction might be responsible for other metabolic disorders such as dyslipidemia and diabetes. While endothelial malfunction appears to be the primary cause, hypertension exacerbates endothelial function deterioration. The heart and cardiovascular system are among the earliest organs affected in the natural history of ADPKD [64]. Several authors have highlighted the relationship between arterial hypertension and cardiac hypertrophy, noting an increased left ventricular mass index (LVMI) in ADPKD subjects with hypertension compared to those with normal BP. Even normotensive ADPKD patients exhibit a higher ventricular mass compared to the healthy population, alongside significant biventricular diastolic dysfunction [66]. Similarly, adult ADPKD patients have been found to have an increased carotid intima–media thickness (cIMT), a subclinical indicator of endothelial damage and a precursor to atherosclerotic plaques that may lead to cardiovascular events such as stroke and myocardial infarction [62,64,65]. Studies comparing ADPKD patients with a healthy population or those with essential hypertension have demonstrated endothelial dysfunction and a significant increase in cIMT, indicating vascular damage [67]. Pulse wave velocity (PWV) is a marker of vascular stiffness and is elevated in hypertensive ADPKD subjects [67]. Atherosclerotic damage is more pronounced in ADPKD patients with hypertension compared to other groups [55,62]. A 2018 study described the prevalence of hypertension and cardiovascular damage in pediatric ADPKD patients: [68]. Among the 21 patients included, central BP was estimated by tonometry and peripheral BP was measured by ABPM. Data collection included LVMI, PWV, and cIMT. A linear correlation was found between central BP and ABPM BP. The percentage of patients with elevated cIMT was higher than of those with elevated LVMI or PWV. The increase in PWV and cIMT precedes the enlargement of LVMI. Linear correlations were found between LVMI and PWV and between LVMI and cIMT. LVMI increase was also correlated with both central BP and ABPM BP. However, no correlation was found between BP levels and cIMT or PWV levels, suggesting that vasculopathy progresses independently of BP in the early stages. In adults with ADPKD, arterial stiffness and hypertrophic vasculopathy are present in the early stages of the disease, even before the onset of hypertension [69,70]. Vascular dysfunction in children with ADPKD is not frequently documented. A study on early vascular damage in ADPKD children compared 15 patients with 15 healthy controls of the same age range and good body mass index (BMI) [71]. Measurements included brachial office BP, central carotid BP, endothelium-dependent dilation via brachial artery flow-mediated dilation (FMD_BA_), and arterial stiffness via PWV. Central BP was significantly increased in ADPKD patients despite normal brachial BP. FMD_BA_ was significantly lower in ADPKD cases than in healthy controls, while PWV was significantly higher. Both FMD_BA_ and PWV were associated with central systolic BP. Intima–media thickness did not differ between the two groups. For all the reasons discussed above, hypertension and left ventricular hypertrophy are the primary factors responsible for the occurrence of early cardiovascular events, which are the leading cause of death in individuals with ADPKD [66]. 

### 1.3. Therapeutic Possibilities of ADPKD in Children

To manage the progression of ADPKD and its renal manifestations in children, several lifestyle and dietary changes are advised. A low-sodium diet helps to manage BP while maintaining adequate hydration and supports overall kidney function. Parents and caregivers should ensure that children avoid smoking and excessive weight gain. Additionally, preventing nephrotoxic drugs is crucial to protect renal health. The cornerstone of pharmacological therapy in pediatric patients with ADPKD is the use of ACE inhibitors (ACEi), such as ramipril. These medications are essential for controlling BP and reducing proteinuria, both of which are significant risk factors for disease progression. In recent years, researchers have explored additional pharmaceutical options. One such option is the use of vasopressin receptor antagonists like tolvaptan. This medication has shown promise in slowing the increase of TKV, a critical factor in ADPKD progression. Vasopressin-mediated cAMP signaling plays a crucial role in cyst proliferation and fluid secretion [54]. Studies in animal models have demonstrated that reducing vasopressin activity can significantly impact cyst development and growth [72]. In a large randomized trial on adult patients, tolvaptan was shown to slow the decline in eGFR and the growth of renal volume [73,74]. However, tolvaptan is currently approved only for the treatment of adults with rapidly progressing disease. Treatment challenges include significant polyuria, thirst, and nocturia. Hepatic impairment is also present in 1.2% of patients; this damage appears to be reversible but requires monitoring of liver function [73]. The first pediatric study on the use of tolvaptan in adolescents is underway [75]. Another group of drugs under study includes mTOR inhibitors like everolimus and sirolimus [76]. These drugs have shown mixed results in clinical trials [77]. While they can reduce kidney volume and slow growth, their overall therapeutic utility remains uncertain. Both mTOR complexes, mTORC1 and mTORC2, are activated in ADPKD [78,79], and inhibiting these pathways has been effective in reducing cyst growth and improving renal function by preventing epithelial cell proliferation, reducing fibrosis, and stimulating apoptosis of lining cells in a murine experimental model [80]. In another study, sirolimus inhibited mTORC1 and renal cyst growth but did not affect renal function deterioration in mice with the *PKD2* gene mutation [78]. However, clinical trials have faced challenges, mainly due to the systemic toxicity and limited renal bioavailability of these drugs [2]. Metformin, a common oral hypoglycemic agent, has also been studied for its potential benefits in ADPKD. It works by activating AMPK, which inhibits both the CFTR (cystic fibrosis transmembrane conductance regulator) and mTOR pathways through the phosphorylation of TSC2 (tuberin), potentially impacting cyst growth and renal function [81]. Further research is needed to understand its efficacy and safety in pediatric patients fully. Managing BP and proteinuria is crucial in ADPKD therapy. Hypertension is a common complication and can significantly worsen kidney function over time. ACE inhibitors (ACEi) and angiotensin receptor blockers (ARBs) are the first-line treatments for managing these conditions. However, these drugs should be used with caution in adolescent females at risk of adolescent pregnancies due to their teratogenic effect even in the first trimester of pregnancy. In children with high–normal BP, ACEi appears to slow the decline in renal function [75]. However, some studies demonstrate that the addition of ARBs to ACEi therapy does not provide any additional benefit [2]. These medications help control BP and reduce proteinuria, thereby preserving renal function and reducing cardiovascular mortality. Managing BP effectively can help reduce the risk of these events. Regular screening and monitoring are vital for managing ADPKD in children. Early detection of hypertension and other complications can significantly impact long-term outcomes. According to the KDIGO guidelines (2019), screening should begin at age 5, with follow-up screenings every three years if no hypertension is detected [2]. Hypertension in children is diagnosed when systolic or diastolic BP is above the 95th percentile for their age, height, and sex. ABPM is recommended for children over five years old to confirm office hypertension diagnoses. Continuous follow-up is essential, with evaluations every 1–3 years to monitor for hypertension and proteinuria. It is also important to check for organ damage in target organs, such as the eyes and heart. Routine abdominal ultrasounds are not necessary unless there are clinical signs of complications like flank pain, gross hematuria, or urinary tract infections. The Network for Early Onset Cystic Kidney Disease (NEOCYST) does not indicate the screening for extrarenal manifestations (cerebral aneurysms, hepatic and pancreatic cysts, cardiac valvulopathies, intestinal diverticula, abdominal wall hernias, and seminal vesicle cysts) unless clinically justified [38]. In summary, managing ADPKD in children requires a comprehensive approach that includes dietary and lifestyle modifications, pharmacological therapy, and regular screening and monitoring. The treatment of hypertension in pediatric patients should adhere to the main pediatric guidelines on the subject, according to age and sex [65]. While ACEi and ARBs remain the cornerstone of treatment, new pharmaceutical options like tolvaptan and mTOR inhibitors offer potential benefits but require further research to establish their safety and efficacy in pediatric patients. In the absence of hypertension and altered renal function in pediatric age, therapeutic indications aim to slow down the decline in renal function by limiting secondary damage. Preventive measures and lifestyle changes are equally important in slowing disease progression and improving long-term outcomes. By providing thorough and continuous care, healthcare providers can help manage the symptoms of ADPKD and enhance the quality of life for affected children.

### 1.4. Psychological Issues

According to the literature, the lives of patients affected by ADPKD are strongly influenced by the course of the disease, which can progress towards renal failure, and by extrarenal cardiovascular, hepatic, pancreatic, and pulmonary manifestations accompanied by pain [8]. Current evidence-based guidelines indicate a multidisciplinary approach to managing ADPKD patients for a comprehensive evaluation of clinical manifestations, complications, prognosis, and the psychosocial impact of the disease on the patient’s life and their family [82]. The issues affecting adult ADPKD patients may include anxiety about the future and employment, family planning, depression, and timing of replacement therapy such as dialysis or transplantation, all accompanied by physical discomforts such as pain and abdominal bloating [39]. For pediatric patients, guidelines regarding psychosocial aspects are controversial: some suggest that presymptomatic screening in children with a family history of ADPKD is not always indicated due to potential negative psychological consequences, while others recommend it. In particular, supportive studies suggest that families of children with ADPKD be encouraged to discuss the risk of disease transmission with their children. Parents affected by inherited diseases often find it challenging to communicate with their children about the genetic condition due to the high psychological burden of so-called “genetic guilt” and seek professional assistance, information about the disease, and guidance on managing their children’s emotional reactions [56,83]. Some studies show that avoiding such discussions can lead to family tensions, misunderstandings, accusations, and secrecy, while open communication with children promotes effective coping strategies, making children more resilient. Genetic guilt issues or fear of the future course of the disease can have a substantial impact on the psychological well-being of young people and families affected by ADPKD. Integrated care should, therefore, include an active investigation of anxieties and sources of psychological support. Finally, it may be helpful to remind parents of their value as positive role models. Various lifestyle interventions may be recommended for ADPKD patients, particularly during adolescence when adequate psychological and nutritional counseling is important. Relevant messages include the importance of a healthy, low-salt diet, adequate fluid intake, and regular physical exercise. Discontinuation of care due to the transition from pediatric to adult nephrology care is a significant risk factor for adverse outcomes in severely affected individuals. Transition guidelines should be followed to prevent loss of medical follow-up. The COVID-19 pandemic has profoundly impacted children and families, with widescale disruptions in individual, family, school, and peer functioning. The impact of the COVID-19 pandemic on pediatric chronic illness groups requires special consideration. Parents can try to be models of optimal psychophysical health and detect the first signs of psychological distress from interactions with their children. However, they could need support from a psychologist to discuss and alleviate the tensions that situations such as the pandemic can trigger. We must understand the challenges and needs of families with chronically ill children and prepare a health and education system that will meet these needs in the future to avoid the risk of physical repercussions and worsening the psychological state of these children [84].

### 1.5. Nutritional Issues

Dietary intervention is an essential component in the treatment of ADPKD to prevent disease progression, limit complications such as hypertension, hyperkalemia, and metabolic acidosis, and prevent malnutrition associated with end-stage renal failure. CKD irreversibly compromises kidney function, preventing the kidneys from eliminating excess waste and other substances, such as phosphorus, sodium, and potassium, accumulating in the body. In advanced cases, it becomes necessary to resort to replacement treatments such as dialysis or transplantation to purify the blood from waste products [8]. To slow down the progression of ADPKD towards renal failure, it is crucial to adopt a dietary style that reduces the workload on the kidneys, preserving their function for as long as possible. For adult patients with advanced-stage ADPKD, there are numerous nutritional guidelines available, while currently, there are no specific guidelines for pediatric patients to prevent and delay renal failure. The objectives of nutritional recommendations include providing adequate calorie intake to maintain nitrogen balance, ensuring a good protein intake relative to the child’s weight, improving fluid and electrolyte balance, lipid and glycemic profile to prevent obesity, optimizing mineral metabolism, and satisfying the patient’s taste preferences. Water is essential for transporting nutrients and oxygen to cells, removing waste products, regulating metabolic processes, maintaining body temperature, and aiding digestion. For children and adolescents, recommended water intake levels were established in 2019 [85]. Quantifying daily water intake in children who lose more fluids through perspiration and have a lower thirst perception is important. For patients with eGFR > 30 mL/min/1.73 m^2^, increased fluid intake is recommended (more than 3 liters per day for adults), which suppresses vasopressin, inhibiting cyst formation and protecting against nephrolithiasis [54]. Control over dietary protein intake is crucial in patients with kidney disease and should be modulated based on the progression of the disease [86]. Reducing protein intake slows down the progression of the disease and controls urea levels, delaying the onset of dialysis. In the early stages of renal failure (eGFR > 60 mL/min/1.73 m^2^), simply normalizing protein intake relative to requirements is sufficient [8]. Energy should primarily be obtained through carbohydrates and lipids. Carbohydrates should constitute 50–60% of daily calories, with simple sugars accounting for less than 15% [86]. Consuming pasta, bread, rice, fruits, sugar-free jams, and juices is preferable, limiting sugar, chocolate, honey, ice cream, sweets, and sugary drinks. Lipids should represent 25–35% of daily calories, eliminating hydrogenated trans fats and limiting whole milk, aged cheeses, cured meats, alcoholic beverages, and spirits. Preferred choices include extra virgin olive oil, fish, and lean meats. Sodium, primarily present in the extracellular space, regulates body fluid volume. The accumulation of sodium and water increases BP. The main sources of sodium are table salt, natural foods (milk, meat, fish, eggs), and processed and preserved foods [86]. In children, sodium intake should be lower than in adults, and for pediatric patients with ADPKD, sodium intake should be lower than recommended for healthy children [85]. A low-sodium diet can be monitored through 24 h urinary sodium excretion, as increased urinary sodium correlates with worsening GFR [87] (Table 1).

Potassium is essential for cellular function, muscle contraction, and cardiac function. As kidney disease progresses, the kidneys become less effective at eliminating potassium, leading to hyperkalemia. Potassium-rich foods include bananas, dried fruits, kiwis, artichokes, spinach, potatoes, cod, trout, prosciutto, salami, speck, bran, oatmeal, and wheat germ [86]. Phosphorus, eliminated by the kidneys, can cause hyperphosphatemia if accumulated, leading to cardiovascular and bone problems. The proper metabolism of phosphorus and calcium, important for bone health, is linked to vitamin D. Therefore, an adequate intake of sodium, potassium, phosphorus, calcium, and vitamin D is essential [88]. There are no established nutritional guidelines for pediatric patients at risk of renal failure. Rather than a strict dietary prescription, it is necessary to provide comprehensive nutritional education accompanying young patients throughout their lives. Nutritional education, along with appropriate lifestyle habits, can delay renal failure and the need for dialysis or kidney transplantation. Educating young patients involves teaching them to recognize protein, sodium, phosphorus, and potassium sources in foods and manage them throughout the day, considering they spend part of the day away from home. The diet should not be overly restrictive to avoid eating behavior disorders but should be manageable for the family of the patient’s age, lifestyle, and family habits. It is helpful to teach children with kidney disease to read food labels, reduce salt intake, avoid using salt at the table, and consume fresh foods while avoiding packaged products. Dietary prescriptions should always be accompanied by nutritional education and shared with the patient and their family. Nutrition in pediatric patients with ADPKD is crucial because the right intake of fluids, protein, sodium, phosphorus, and potassium can delay the progression towards chronic renal failure, avoid replacement therapies, and improve the patient’s quality of life.

## 2. Summary

**Figure 1:**
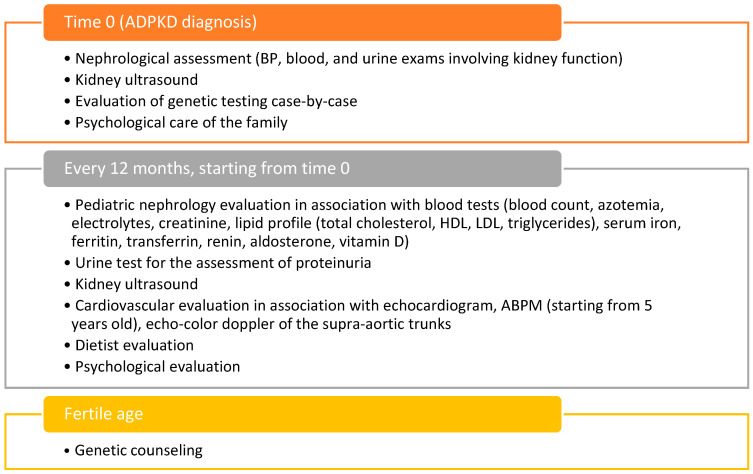
Proposal of flow-chart of 360-degree management of a child with ADPKD. BP: blood pressure; HDL: high-density lipoproteins; LDL: low-density lipoproteins; ABPM: ambulatory blood pressure monitoring.

ADPKD is a genetic disorder that results in the formation of multiple cysts in the kidneys, potentially leading to renal failure. Although commonly presenting in adulthood, ADPKD can also affect children, necessitating early and thorough management to prevent or delay complications. A multidisciplinary approach is essential for effectively managing children with ADPKD, as it ensures comprehensive care addressing the various dimensions of the disease. ADPKD impacts multiple organ systems beyond the kidneys, including the liver, cardiovascular system, and gastrointestinal tract. Effective management requires collaboration among various healthcare professionals. Nephrologists are central to this team, as they monitor kidney function, control BP, and guide lifestyle changes to slow disease progression. Pediatricians oversee overall health, ensuring average growth and development and addressing general health concerns. Cardiologists play a crucial role due to the increased risk of hypertension and cardiovascular issues, monitoring heart health and managing related conditions. Geneticists provide essential genetic counseling, helping families understand the hereditary nature of ADPKD and aiding in family planning and decision-making. Nutrition is another critical aspect, with dietitians helping to design kidney-friendly diets that support overall health and manage symptoms. Additionally, chronic illnesses like ADPKD can impact mental health, so psychologists provide support to children and their families, helping them cope with the emotional and psychological challenges of the disease. Starting from a previous model [89] and the consensus by Gimpel et al. [38], we propose a follow-up for children with ADPKD. Regular follow-up is vital for children with ADPKD to monitor disease progression, manage symptoms, and adjust treatment plans as needed. Early detection of complications allows for timely interventions, significantly improving outcomes. A proposed timeline for follow-up includes comprehensive reviews by a pediatric nephrologist every three to six months, including BP monitoring, renal function tests, and ultrasound imaging to track kidney size and cyst development. Annually, cardiologist evaluations monitor for hypertension and other cardiovascular issues, while pediatricians conduct general health check-ups, including growth and development assessments. Dietary assessments by a dietitian are recommended to meet nutritional needs and make necessary dietary adjustments. The timing of dietary evaluation should be scheduled according to the individual patient’s needs, at least once a year. Psychological support sessions are essential, particularly during stressful periods such as starting school or adolescence (Figure 1).

In conclusion, the multidisciplinary management of children with ADPKD is crucial for addressing the complex needs of these patients. Regular follow-ups and a comprehensive, individualized care plan can significantly enhance quality of life and slow disease progression. By leveraging the expertise of various healthcare professionals, children with ADPKD can receive optimal care and support throughout their development.

## 3. Future Directions

In the short term, it is important to promote clinical studies on drugs already in use for adults, such as tolvaptan, to establish their safety and efficacy in the pediatric population. Other objectives include determining the significance of *PKD1* mutations through bioinformatic analyses, family studies, in vitro tests, and animal studies to improve molecular diagnoses and prognoses. Cataloguing genetic variants beyond the ADPKD genes that influence the phenotype may also have prognostic value. Future research should focus on identifying triggers and understanding the exact pathways that regulate key cellular transitions. Additionally, gaining a better understanding of the stimuli that promote cyst growth could reveal interesting targets for therapeutic interventions. In the cardiovascular domain, remote monitoring technologies can enhance the management of hypertension and cardiac complications management. Wearable devices and telemedicine applications can facilitate continuous and real-time monitoring, allowing timely interventions. Furthermore, education and psychosocial support for families need to evolve, incorporating virtual support programs and digital resources to better manage the stress and anxiety associated with chronic disease. Research into innovative dietary interventions may provide new strategies to slow disease progression through nutrition. The integration of multidisciplinary and personalized approaches, supported by advanced technologies and new scientific discoveries, represents the key to significantly improving the management of ADPKD in the pediatric population in the near future.

## 4. Conclusions

ADPKD, a genetic disorder causing multiple kidney cysts, can lead to renal failure and affect children, necessitating early management to prevent complications. A multidisciplinary approach is crucial for comprehensive care, involving various specialists. Nephrologists monitor kidney function and manage BP, while pediatricians ensure overall health and development. Cardiologists address cardiovascular risks, and geneticists provide counseling on the hereditary nature of ADPKD. Dietitians design kidney-friendly diets, and psychologists offer support for the mental health challenges associated with chronic illness. Regular follow-ups are essential, with pediatric nephrologist reviews every three to six months to monitor disease progression and annual cardiologist evaluations. Adequate psychological and social support can help manage the stress and anxiety associated with chronic disease, promoting overall well-being. Dietary aspects also play a key role in the management of ADPKD. An appropriate diet, low in salt and protein but rich in fruits, vegetables, and fiber, can help control BP and preserve renal function. It is crucial to educate patients and their families on the importance of proper nutrition as an integral part of treatment. In conclusion, a multidisciplinary approach that integrates genetic, renal, cardiovascular, psychosocial, and dietary interventions is essential to improve the management and clinical outcomes of ADPKD in pediatric patients. Ongoing research and implementing new therapeutic strategies are key to addressing the challenges posed by this complex disease. 

## Figures and Tables

**Table 1:** LARN for sodium in the pediatric population (modified from [85]).

	Age	Adequate Intake (AI) g/Day
Infants	6–12 months	0.4
Children– Adolescents	1–3 years	0.7
	4–6 years	0.9
	7–11 years	1.1
	11–17 years	1.5

LARN: National Recommended Energy and Nutrient Intake Levels.

## Data Availability

Not applicable.
